# Designing a Multiplex PCR for Rapid and Accurate Detection of Metallobetalactamases Resistant Genes from *Acinetobacter baumanii*Isolates in Tehran City, Iran

**DOI:** 10.30699/IJP.2023.2007142.3144

**Published:** 2023-10-15

**Authors:** Zahra Mottaghiyan, Davoud Esmaeili, Mohammad Hossein Ahmadi, Mohammad Niakan

**Affiliations:** 1 *Department of Microbiology, Faculty of Medicine, Shahed University, Tehran, Iran*; 2 *Baqiyatallah University of Medical Sciences, Tehran, Iran*

**Keywords:** Acinetobacter baumannii, MBL genes, Multiplex PCR

## Abstract

**Background & Objective:**

*Acinetobacter baumannii* strains harboring Meallobetalactamases (MBL) pose a significant threat in the context of nosocomial infections. The present investigation was undertaken with the objective of devising a Multiplex PCR methodology for the concurrent detection of MBL genes within A. *baumannii* strains prevalent in Tehran City, Iran.

**Methods:**

Between October 2020 and February 2021, 100 strains of *A. baumannii* were procured from burn specimens of hospitalized patients at Motahhari Hospital in Tehran. The identification of *A. baumannii* strains involved conventional biochemical techniques, coupled with confirmation of the presence of the bla _OXA-51_ gene. Antibiotic susceptibility was assessed using the Kirby–Bauer disc diffusion test. MBL-producing strains were characterized through a phenotypic approach employing the combined disk test, alongside Multiplex PCR for the simultaneous identification of bla _VIM_, bla _IMP_, bla _GIM_, and bla _NDM_ genes. Statistical analyses were conducted using the chi-square test, with SPSS version 20.0 employed for data processing.

**Results:**

Among 100 strains examined, 96.1% exhibited positivity for MBL, as determined by the combined disk test. The study revealed a predominance of extensively drug-resistant (XDR) strains, with colistin demonstrating the highest level of sensitivity. The genotypic assay unveiled that Multiplex PCR identified bla _VIM_, bla _NDM_, and bla _IMP_ in 20 strains, bla _VIM_ and bla _NDM_ in 30 strains, and exclusively the bla NDM gene in 45 strains. Notably, the Multiplex PCR technique exhibited the capacity to concurrently detect MBL genes (bla _VIM_, bla _IMP_, bla _GIM_, bla _NDM_) in 2 strains.

**Conclusion:**

The current investigation underscores prevalence of the bla _NDM_ gene within clinical strains of *A. baumannii*. Furthermore, Multiplex PCR emerges as a robust and highly sensitive technique for rapid discernment of the MBL genes within in *A.* *baumannii* strains.

## Introduction

*Acinetobacter baumannii* is a life-threatening and significant opportunistic pathogen commonly found in hospitals (1). Its inherent capabilities including survival in challenging environmental conditions and acquisition of antibiotic resistance mechanisms (2, 3), have resulted in the emergence of multidrug resistance (MDR), extensive drug resistance (XDR), and even PDR phenotypes, posing considerable challenges to healthcare systems and the health community. Notably, the ability to develop resistance to carbapenems, which are broad-spectrum β-lactam antibiotics, is a critical characteristic of *A. baumannii* (4, 5).

The carbapenems class are the most effective antibiotics for treatment of *A. baumannii* infections. The primary mechanism for carbapenem resistance involves the production of β-lactamase enzymes (6, 7). In *A. baumannii*, four molecular classes of β-lactamase (A, B, C, and D) have been identified (8). Among these, Class B β-lactamases, known as Metallo-β-lactamases (MBL), need zinc ions for their catalytic activity (9). MBLs exhibit the ability to hydrolyze all beta-lactam classes except monobactams (10). Examples of MBLs in *A. baumannii *include*bla*_IMP_, *bla*_VIM_, *bla*_GIM_, *bla*_DIM_, *bla *_SPM_, *bla*_SIM_, and *bla *_NDM_ that *bla *_IMP_ and *bla*_VIM_ allelic variants as predominant MBLs globally (10, 11).

 Infectious diseases caused by MBL-producing organisms are associated with elevated mortality and morbidity rates (12). In recent years, due to the Sars-Cov-2 pandemic, there has been a surge in the prevalence of MDR, XDR, and even PDR *A. baumannii* infections, posing significant challenges for hospitalized patients. Therefore, utilization of rapid diagnostic methods, such as Multiplex PCR, enables simultaneous detection of the drug resistance genes, aiding physicians in selecting appropriate antibiotics. The current study represents the first attempt to design a multiplex PCR method for the concurrent detection of MBL genes (bla _VIM_, bla _IMP_, bla _GIM_, bla _NDM_) from *A. baumannii* strains isolated in Tehran, Iran.

## Material and Methods

**Sampling and Isolation of** ***Acinetobacter baumannii*** **Specimens**

In this cross‑sectional investigation, a total of 100 clinical *A. baumannii* strains were obtained from burn samples of patients admitted to the Motahhari Hospital in Tehran city. Samples were collected from various clinical sources in the microbiology laboratories of the Motahhari hospital (Table 1). All *A. baumannii* strains were collected between October 2020 and February 2021. Samples collected from body sites other than burn wonds, and those lacking phenotypical resistance, were excluded from the study. All strains were sub‐cultured on EMB agar and Blood agar, followed by an incubation period of 18-24 hours at 37°C under aerobic conditions. This study was approved by the Research Ethics Committee of Shahed University (Ethical code: IR.SHAHED.REC.1300.064).

**Table1:** Prevalence of *A. baumannii* isolated from the Motahhari Hospital patients to type of infections

Frequency (%)	Sample type
**25**	**Sputum**
**26**	**Urine**
**5**	**Catheter**
**12**	**Trachea**
**10**	**Wound**
**5**	**Blood**
**7**	**Cerebrospinal fluid(CSF)**
**1**	**Respiratory tract**

**Identification of** ***Acinetobacter baumannii*** **Strains**

For the initial identification of *A. baumannii* strains, conventional biochemical techniques including growth on MacConkey agar, motility, sugar fermentation, triple sugar iron (TSI), colony morphology, positive catalase, citrate, negative oxidase, growth at 44°C, and sulfide indole motility (SIM) were employed. The *bla*_OXA-51_ gene was used to validate the strain identification. The PCR method was used to detect the *bla*_OXA-51_ gene in *A.* *baumannii* isolates using specific primers (Table 2). PCR conditions were consistent with those of the Multiplex PCR. Each verified strain was inoculated into a vial containing Brain Heart Infusion broth with 20% glycerol and stored at - 80°C.

**Antimicrobial Susceptibility Testing**

Antimicrobial susceptibility was assessed by conducting a Kirby-Bauer disc diffusion test with a 0.5 McFarland bacterial suspension on Mueller Hinton agar. In accordance with CLSI recommendations (13), nine antibiotic discs were utilized to evaluate isolates for MDR and XDR using ampicillin (10 μg), cefotaxime (30 μg), ceftazidime (30 μg), imipenem (10 μg), ciprofloxacin (5 μg), amikacin (30 μg), gentamicin (10 μg), cefepime (30 μg), and colistin (10 μg). *Escherichia coli* ATCC 25922 and *A. baumannii* ATCC 19606 were employed as negative and positive controls, respectively. 

**Phenotypic Detection of the MBL-producing Strains by Combined Disc Test**

Initially, a bacterial suspension was prepared based on the 0.5 McFarland standard and streaked on Muller Hinton agar medium. The beta-lactam disc was immersed in 0.5 M EDTA, placed on the Muller Hinton agar medium, and positioned alongside the beta-lactam disc alone. Following an 18-hour incubation at 35°C, the diameter of the inhibition zone around the discs was measured. If the diameter around the imipenem/EDTA disc compared to imipenem alone increased by 7 mm or more, it indicated the presence of the metallobetallactamase enzyme.

**Multiplex PCR for Detection of** ***bla***_VIM, _***bla***_IMP_**, *bla***_GIM _**and** ***bla***_NDM_** Genes **

The MBL gene sequences were initially verified on the NCBI website. Primers were designed using the Genscript program. Results of the blasting of the Forward and Reverse primers (https://blast.ncbi.nlm.nih.gov/Blast.cgi) indicated the suitability of the proposed primers. Primers were subsequently tested in an online in silico PCR amplification program after evaluation by the Oligo Analyzer software for specifically generated primers (http://insilico.ehu.es). As a result, specific primers for VIM, IMP, GIM, and NDM (Table 2), were employed to amplify the genes using multiplex PCR. Various genome dilutions were used to assess PCR sensitivity. Sequential dilutions of nucleic acids were created, and subsequent genomic dilutions underwent PCR. The dilution at which PCR results were obtained at the lowest concentration was considered. PCR reactions were carried out on the nucleic acids of *Bacillus subtilis* and *Staphylococcus aureus* to establish primer specificity. Genomic DNA was extracted using the Bioneer kit (Bioneer, South Korea), and the concentration of collected DNA was measured using Nanodrop spectrophotometry. The MBL genes *bla*_VIM,_ *bla*_IMP_, *bla*_GIM_, and *bla*_NDM_ were identified using the multiplex PCR approach. The reaction mixture comprised template DNA, Master mix (cat. No. 180301-50), and Forward/Reverse primers. The total reaction volume was 25 μL. Amplification conditions for *bla*_VIM,_ *bla*_IMP_, *bla*_GIM_, and *bla*_NDM_ included an initial denaturation for 5 minutes at 94°C, followed by 35 cycles at 94°C for 40 seconds, annealing at 60ºC for 25 seconds, extension at 72°C for 1 minute, and a final extension at 72°C for 5 minutes. Agarose gel electrophoresis at 1% (w/v) was conducted at 80 V for 60 minutes in 1X TBE, followed by multiplex PCR analysis to confirm the presence of MBL genes.

**2.6. Statistical Analysis**

Data were analyzed using a Chi-square test using SPSS software version 20.0 (SPSS Inc., Chicago, IL., USA). A P-value < 0.05 was considered statistically significant.

**Table 2:** Primers are used in amplification of the selected genes

Gene	Primer	Nucleotide sequence	Amplicon size (bp)	Tm(°C)
IMP	F	TCCAGAACCTTGACCGAACG	466	60
IMP	R	CACGCTCCACAAACCAAGTG	466	60
GIM	F	CAGACAAGCTGTGACCGTCT	928	60
GIM	R	GACTATCGTCGCCGCACTTA	928	60
VIM	F	CCACTGCGATCCCCGGAAAA	232	63
VIM	R	ACAGGCCAGCCATTACGCTC	232	63
NDM	F	CGGCACCGACATCGCTTTTG	160	63
NDM	R	GGCGGAATGGCTCATCACGA	160	63
*bla*_OXA-51_	F	AGGACATGACCCTAGGCGAT	166	60
*bla*_OXA-51_	R	AAAGGACCCACCAGCCAAAA	166	60

## Results

**Characteristics of the Clinical Strains**

In this study, 100* A. baumannii* strains were gathered from different clinical sources in burn patients of Motahhari Hospital as follows: sputum 25%, urine 26%, trachea 12%, wound 10%, cerebrospinal fluid (CSF) 7%, catheter 5%, blood 5%, and respiratory tract 1% (Table 1). 

**Antimicrobial Sensitivity Testing**

**Fig. 1:**
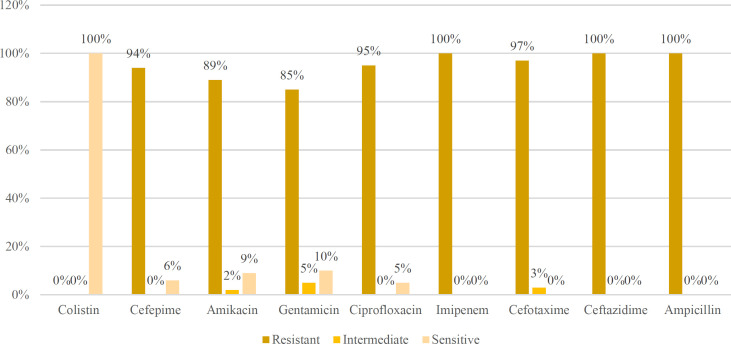
Antibiotic susceptibility testing of 100 *A. baumannii* isolates against a panel of 9 Antibiotics

The Kirby-Bauer disc diffusion technique was used to test all 100 *A. baumannii* strains on Mueller-Hinton agar against a panel of 9 antibiotic discs. The findings were then analyzed by CLSI recommendations (13). Numerous isolates were XDR (Figure 1). The proportion of strains that were resistant to ampicillin (100%), ceftazidime (100%), and imipenem (100%) was notably high. Additionally, the percentage of antibiotic resistance among isolates to other antibiotics was as follows: cefotaxime (97%), ciprofloxacin (95%), cefepime (94%), amikacin (89%), and gentamicin (85%). According to the results, *A. baumannii* strains exhibited higher sensitivity (100%) to colistin.

**Phenotypic Detection of MBL-Producing Strains (Combined Disc Test)**

The Combined Disk Test method was employed to determine MBL-positive isolates under phenotypic conditions. If the difference between the inhibition zones of the imipenem/EDTA disc and imipenem disc was ≥7mm, the combined disk test was interpreted as positive for MBL. Detection of Metallo β-Lactamase in phenotype conditions elucidated that 96.1% of strains could produce MBL.

**Detection of** ***bla***_VIM_**, *bla***_IMP_**, *bla***_GIM_**, and *bla***_NDM_** Genes**

 Detection of the MBL (*IMP*, *VIM*, GIM, *NDM*) genes with specially designed primers by multiplex PCR is presented in Table 3. To designate sensitivity of the MBL primers, serial dilutions ranging from 10^-1^ to 10^-16^ were used. The lowest dilution at which the PCR result was positive was considered as PCR sensitivity. Moreover, a significant correlation (*P*<0.05) between Ciprofloxacin (5 μg) and NDM was observed (Table 4).

**Table 3:** Frequency of the MBL genes in 100 XDR *Acinetobacter baumannii* strains

Genes	Frequency (%)
*bla *_IMP_ *, bla *_VIM_ *, bla *_GIM_ *, bla *_NDM_	2
*bla *_IMP_ *, bla *_VIM_ *, bla *_NDM_	20
*bla *_VIM_ *, bla *_NDM_	30
*bla *_NDM_	45

**Table 4:** Relation between antimicrobial agents and gene frequency in *A. baumannii* strains (R= Resistance, I= Intermediate, S= Sensitive, P= Positive, N= Negative)

NDM			GIM			IMP			VIM			Antibiotics			
**N**	**P**		**N**	**P**		**N**	**P**		**N**	**P**	
**-**	-		-	--		-	-		-	-		S		**Cefotaxime (30μg)**	
**0**	3		3	0		2	1		1	2		I			
**1**	96		96	1		75	22		45	52		R			
**0.86**			0.86			0.66			0.65			p-v			
**1**	4		5	0		5	0		1	4		S		**Ciprofloxacin (5μg)**	
			-	-		-	-		-	-		I			
**0**	95		94	1		72	23		45	50		R			
**0.00**			0.81			0.21			0.23			p-v			
**0**	10		10	0		7	3		6	4		S		**Gentamicin (10μg)**	
**0**	5		5	0		4	1		2	3		I			
**1**	84		84	1		66	19		38	47		R			
**0.91**			0.91			0.85			0.63			p-v			
**0**	9		9	0		5	4		6	3		S		**Amikacin (30μg)**	
**0**	2		2	0		2	0		0	2		I			
**1**	88		88	1		70	19		40	49		R			
**0.93**			0.93			0.21			0.19			p-v			
**0**	6		6	0		3	3		3	3		S		**Cefepim (30μg)**	
			-	-		-	-		-	-		I			
**1**	93		93	1		74	20		43	51		R			
**0.8**			0.8			0.1			0.83			p-v			

## Discussion

*Acinetobacter baumannii*, identified as a critical nosocomial pathogen, poses a significant threat to human health. MBLs represent the most effective β-lactamases capable of hydrolyzing all β-lactams, with the exception of monobactams, and remain unhampered by any known inhibitor (14, 15). Given the escalating prevalence of MBL-producing strains, swift diagnostic techniques, notably molecular methods, are imperative for promptly ascertaining MBL resistance profiles in hospitalized strains. This approach facilitates the management of nosocomial infections and ensures timely and appropriate therapeutic interventions. The indiscriminate application of antibiotics coupled with prolonged hospital stays contributes to the proliferation of antibiotic resistance among *A. baumannii* strains (16).

Numerous investigations have documented prevalence of the MDR and XDR strains of *A. baumannii*, with the majority exhibiting sensitivity solely to colistin. Our findings align with those of Soltani *et al.,* (17), who reported a 92.4% incidence of XDR strains in their study. Asadian *et al.,* (18) observed 100% XDR prevalence among their 79 strains. In 2019, Girija *et al.,* (19) noted rates of 71.23% MDR and 39.72% XDR. Another study reported MDR and XDR frequencies of 83.9% and 16.1%, respectively, with colistin-resistant *A. baumannii* identified in 7.6% of cases. In spite of these findings, Monfared concluded that colistin remains the most efficacious treatment option presently available (20).

Our investigation demonstrated that 96.1% of isolates exhibited phenotypic MBL production, as determined by the Combined Disc Test. A comparable study by Saleh *et al.,* investigated MBL phenotypic detection via the Combined Disc Test in 52 imipenem-resistant A. *baumannii* strains, revealing that 90.4% produced MBL (21). In a 2022 Indian study, 70.5% of carbapenem-resistant A. *baumannii* strains were identified as MBL producers (CDT positive). Rouf *et al.,* underscored the cost-effectiveness and routine applicability of combined disk tests and other phenotypic assays for carbapenemase producers. However, for a comprehensive assessment, they advocated exploration of the resistance genes through genotypic methods (22).

In our study, specific primers were employed for simultaneous and rapid detection of the MBL genes, encompassing *bla*_VIM_, *bla*_IMP_, *bla*_GIM_, and *bla*_NDM_. The outcomes evinced concordance between phenotypic and genotypic tests for clinical *A.baumannii* strains, affirming the 100% specificity of the designed primers. While numerous studies have detected MBL genes in *A. baumannii* isolates using PCR, only a minority have undertaken simultaneous identification of these genes. Given the high prevalence of antibiotic resistance in *A.baumannii* strains in Iran, we opted for designed primers with unerring specificity for simultaneous MBL gene detection. Ranjbar *et al*., employed a multiplex PCR approach targeting *bla*_OXA-48_, *bla *_NDM_, and *bla*_OXA-23_ genes, and reported reasonable specificity for concurrent MBL gene detection in *A. baumannii* strains (23). Massik *et al*., identified MBL genes, including *bla*_IMP_, *bla*_OXA-23_, *bla*_VIM_, and *bla*_OXA-51_, via multiplex PCR. They ascertained presence of *bla*_OXA 51_ gene in all strains, while *bla *_OXA 23_ gene was detected in 53 isolates (91%). However, MBL genes were not identified using multiplex PCR. In light of these discordant genotypic and phenotypic results, the authors posit two potential explanations: firstly, EDTA may serve as a source of false positives, as prior studies have suggested its permeabilizing effect may enhance membrane sensitivity in Gram Negative Bacteria (GNB); secondly, a more comprehensive exploration of additional MBL genes would have facilitated a more accurate conclusion (24). In a study by Raieszadeh *et al*., primers targeting ESBL genes (*KPC*, *AMPC*, *TEM*) were able to concurrently detect positive control samples through multiplex PCR. Raieszadeh concluded that rapid identification methods are imperative for a precise and swift detection of antibiotic resistance genes. Diverse factors, including improper usage of antibiotics, prolonged hospitalization, sample type, divergent diagnostic methodologies for gene identification, geographical variables, as well as patient gender and age, account for variability in the research outcomes (25, 26)

## Conclusion

This study showed that the Multiplex PCR method, boasting 100% specificity, offers a reliable means of detecting MBL genes in *A.* *baumannii* isolates. The findings of this study, in conjunction with other pertinent research, underscore an escalating trend in antibiotic resistance among clinical strains of *A.* *baumannii*. The temporal alignment of patient sampling with the Sars-Cov-2 pandemic led to heightened usage of broad-spectrum antibiotics, consequently exacerbating resistance to conventional antibiotics in hospital strains of *A. baumannii*. 

## Funding

No funds, grants, or other support was received.

## Ethics approval:

This study was performed in line with the principles of the Declaration of Helsinki. Approval was granted by the Ethics Committee of University B (July 27, 2020/ IR.SHAHED.REC.1399.064).

Informed consent was obtained from all individual participants included in the study.

## Conflict of Interest

The authors have no competing interests to declare that are relevant to the content of this article.
